# Propionic Acid and Sodium Benzoate Affected Biogenic Amine Formation, Microbial Community, and Quality of Oat Silage

**DOI:** 10.3389/fmicb.2021.750920

**Published:** 2021-11-08

**Authors:** Tingting Jia, Ying Yun, Zhu Yu

**Affiliations:** ^1^College of Animal Science and Technology, China Agricultural University, Beijing, China; ^2^Institute of Grassland Research, Chinese Academy of Agricultural Sciences, Hohhot, China; ^3^College of Grassland Science and Technology, China Agricultural University, Beijing, China

**Keywords:** additives, biogenic amine, microbial community, oat silage, quality

## Abstract

Investigating the microbial communities and biogenic amine (BA) formation in silage is of vital for improving the quality and safety of oat silage. The present study evaluated the effects of propionic acid (P) and sodium benzoate (SB) on the quality properties, microbial communities, and BA formation in oat silage. Oat was harvested at boot stage and ensiled using P and SB as additives in mini silos, followed by 14 days of aerobic exposure. The results showed that P and SB improved fermentation quality of oat silage, increased the lactic acid content, and decreased pH value and ammonia nitrogen content. Putrescine, cadaverine, and tyramine were the dominant BAs in oat silage; spermidine and spermine were not detected. The control silage had the highest content of total biogenic amine (TBA, 2506.7 mg kg^–1^ DM), and decreased by 51.1 and 57.7% after adding P and SB, respectively. Moreover, a lower putrescine, cadaverine, and tyramine content and undesirable microbes, such as *Caproiciproducens*, *Stenotrophomonas*, *Herbinix*, and *Enterobacter* genera, were observed in P and SB silages, which was beneficial for oat silage quality. The fungal community of P silage was dominated by *Monascus fuliginosus*, and the temperature, pH and ammonia nitrogen content increased after exposure to air. *Sedimentibacter*, *Herbinix*, *Caproiciproducens*, *Enterobacter*, and *Escherichia-Shigella* were found to be positively correlated with BA formation in oat silage. Overall, P and SB effectively inhibit the undesirable microbes and BA formation in oat silage, the P silage exhibited lower aerobic stability than the SB silage.

## Introduction

Ensiled whole plant oats are an important feedstuff for ruminants in large parts of the world. Oat silage is rich in dietary fibers, minerals, vitamins, and phytochemicals beneficial to animal health (Kirilov, 2004). The quality and safety of silage are paramount and correlate with the fermentation pattern (Gollop et al., 2005). In recent years, increasing attention has been given to silage safety. A fermentation process prevents forage deterioration and extends its preservation time since the organic acids released by lactic acid bacteria (LAB) inhibit the growth of undesirable microorganisms. The succession growth of LAB and their metabolic activities are essential for control the quality and safety of silage (Driehuis et al., 2018). However, the fermentation of oat silage is a complex biochemical process that includes interactions between various microorganisms (Duniere et al., 2017; Li et al., 2021). The activity of many microbes in silage may cause an extensive breakdown of amino acids into biogenic amines (McDonald and Whittenbury, 1973; Scherer et al., 2015).

Biogenic amine (BA) is a basic organic N-containing compound with low molecular weight (Maijala et al., 1993). BA arises from the decarboxylation of amino acids by microorganisms during silage fermentation (Nishino et al., 2007; Scherer et al., 2019). BAs are common in silage; most BAs that occur during ensilage are tryptamine, β-phenylethylamine, putrescine, cadaverine, histamine, tyramine, spermine, and spermidine (Steidlová and Kalač, 2004; Nishino et al., 2007; Selwet et al., 2013; Guo et al., 2018; Scherer et al., 2019). The BA content in silage is much higher than that in fresh forage, dried feed, concentrate, or well-conserved animal protein feed (Phuntsok et al., 1998; Scherer et al., 2015). The presence of high levels of BA during ensilage may pose a potential safety hazard to animals (Saarinen, 2002; Driehuis et al., 2018). Many liver and kidney diseases in animals originate from the detoxification and catabolism of BA (Křížek, 1993). Substantial amounts of BA accumulation in silage have been linked to an increased risk of undesirable changes (Křížek, 1991; Scherer et al., 2015) and are implicated as being responsible for low dry matter intake of livestock (Van Os et al., 1997; Saarinen, 2002). Tyramine and histamine may harm the rumen mucosa and ruminal microorganisms (Steidlová and Kalač, 2002). Thus, evaluating the BA in silage is particularly valuable for the quality and safety of silage and is helpful in assessing potential health risks for animals.

The type and amount of BA formed in silage depend on various factors, including forage maturity stage, dry matter concentration, fermentation temperature, hygiene during harvest, and additive usage (McDonald et al., 1991). It has been reported that additives greatly influence BA production in silage (Steidlová and Kalač, 2004; Nishino et al., 2007; Selwet et al., 2013; Guo et al., 2018; Scherer et al., 2019). In fact, after fermentation, microorganisms present in the silage may play a central role in the formation of BA. Many microorganisms such as *Bacillus*, *Clostridium*, *Enterobacteriaceae*, and some yeasts are capable of excreting amino acid decarboxylases to produce BA (Scherer et al., 2015), and the microbial community in silage may affect the BA content. Characterizing the microbial community and evaluating the BA distribution and content is beneficial for maintaining high quality and ensuring the safety of oat silage.

Additives have been widely used to improve the quality of silage. Propionic acid (P) and sodium benzoate (SB) are common additives with antimicrobial properties that are often used as silage preservatives (Queiroz et al., 2018). Although many studies have reported the effects of P and SB on the fermentation quality and aerobic stability of silage (Kleinschmit et al., 2005; Chen et al., 2014; Zhang et al., 2018; Larissa et al., 2021), little is known about their effect on BA formation in oat silage. Furthermore, few studies have focused on BA formation in oat silage, and the relationship between BA and the microbial community in oat silage is still not well known. This study aimed to investigate the BA types and levels and microbial communities in oat silages that were treated with P and SB. Pearson correlation analysis was performed to clarify the correlations between BA and microbial communities.

## Materials and Methods

### Ensiling

Oat (*Avena sativa* L.) was grown at a farm in Shaogen town, Ar Horqin Banner, Chifeng City, Inner Mongolia Autonomous Region. The whole-crop oat forage was harvested early in the morning at the boot stage (October 2018). Oat forage was wilted on a polyethylene sheet for approximately 24 h. After wilting, the dry matter content of oat forage was 254.5 g/kg fresh matter (FM). Then the oat forage was chopped to a 1–2 cm theoretical length for silage preparation. The chopped oat forage was treated with propionic acid (P) and sodium benzoate (SB). P and SB were applied at 4.0 g/kg FM. The two additives were dissolved in deionized water, and 10 ml of solution per kg of oat forage was applied using a manual sprayer. The control silage was sprayed with an equivalent volume of deionized water.

Following treatment application, forages were mixed well with additives, packed into 3 L plastic silos equipped with a lid that only enabled gas release, with a density of 700 kg/m^3^. All silos were sealed and stored at ambient temperature (25°C ± 2°C). Triplicate silos for each treatment were opened after 210 days of ensiling. Prior to ensiling, samples from each treatment were collected for dry matter and biogenic amine (BA) analysis. At sampling, triplicate mini silos were opened, and the contents were thoroughly mixed by hand. Sub-samples were then collected for dry matter, fermentation quality, BA content, and microbial community analysis.

### Aerobic Stability Measurement

After sampling, the silos were subjected to an aerobic stability test according to Zhang et al. (2018). About 1.5 kg of each silo was placed into separate 3 L plastic containers and stored at room temperature (25°C ± 2°C). Two layers of cheesecloth were placed over each container to prevent drying and dust contamination and allowed the air to penetrate (Zhang et al., 2018). The room and silage temperatures were monitored every 30 min using a data logger (Campbell CR3000). The thermocouple probes were inserted into each container at the geometric center of the silage mass. Aerobic stability was defined as the time taken to increase the sample temperature by 2°C above room temperature (Moran et al., 1996). In addition, the aerobic silage samples were thoroughly mixed and sampled after 7 and 14 days of aerobic exposure to analyze the fermentation quality property, BA content, and microbial community.

### Dry Matter Content and Fermentation Quality Property Analysis

About 200 g of pre-ensiled material, terminal silage and aerobically exposed silage samples were dried at 65°C for 48 h by oven to measure the DM content. The terminal silage and aerobically exposed silage samples (20 g) were mixed in 180 ml of distilled water and homogenized in a juicer for 2 min. Then, the mixture was filtered through two layers of cheesecloth and one layer of qualitative filter paper. The filtrate was used to determine pH, organic acid, and ammonia nitrogen levels. The pH was measured using a pH meter (PHS-3C, INESA Scientific Instrument Co., Ltd., Shanghai, China). The organic acid (lactic acid, acetic acid, propionic acid, and butyric acid) levels were determined using high-performance liquid chromatography (HPLC) [column: Shodex RS pak KC-811, Showa Denko K.K., Kawasaki, Japan; detector: diode array detector (DAD), 210 nm, SPD-20A, Shimadzu Co., Ltd., Kyoto, Japan; eluent: 3 mmol/L HClO_4_, at a flow rate of 1.0 mL/min; column temperature: 50°C]. The ammonia nitrogen content was analyzed using the phenol and sodium hypochlorite method (Broderick and Kang, 1980).

### Biogenic Amine Analysis

The BA content was determined based on the method described by Nishino et al. (2007) with slight modifications. Lyophilized samples of oat, terminal, and aerobically exposed silage were used to determine the contents of eight BAs (tryptamine, β-phenylethylamine, putrescine, cadaverine, histamine, tyramine, spermidine and spermine). Trichloroacetic acid (25 mL, 50 g/L) was added to 2.5 g lyophilized powder samples and extracted for 60 min. After centrifugation for 10 min at 1,800 × *g*, the supernatant was filtered through a layer of filter paper. The remnant was then extracted with trichloroacetic acid, as described above. The volume of the filtrate was adjusted to 25 ml using 50 g/L trichloroacetic acid. One milliliter of the extract was placed in a 5 ml volumetric flask. Sodium hydroxide (2 N, 200 μl), saturated sodium bicarbonate (300 μl) and dansyl chloride solution (10 mg/ml, 1 ml) were added to the extract. After incubation at 40°C for 45 min in the dark, the reactant was mixed with 100 μl of 25% ammonium hydroxide and incubated at ambient temperature for 30 min to remove the residual dansyl chloride. After that the volume of the reaction mixture was adjusted to 5 ml with acetonitrile. Finally, the mixture was centrifuged for 5 min at 10,000 × *g*. The supernatant was then filtered through a 0.22 μm syringe filter and subjected to high-performance liquid chromatography (HPLC).

Separation was carried out on a C_18_ column (ReproSil-Pur Basic, 5 μm, 250 × 4.6 mm, Dr. Maisch GmbH, Germany) with a DAD. Gradient elution was performed with acetonitrile (solvent A) and 0.1 mol/l ammonium acetate (solvent B). The gradient elution procedure was as follows: 50% A:50% B at 0.01 min, 90% A:10% B at 25 min, 90% A:10% B at 35 min, and 50% A:50% B at 45 min. The elution flow rate was 0.8 ml/min, the column temperature was 30°C, the wavelength was 254 nm, and the sample injection volume was 20 μl.

### Microbial Community Analysis

The microbial total DNA extraction of the oat silage samples was performed according to the method of Zheng et al. (2017). Twenty grams of sample was collected, mixed with 80 ml of sterile water, and stirred at 120 rpm and 4°C for 2 h. The samples were filtered through two layers of sterile gauze and then centrifuged at 10,000 × *g* for 15 min at 4°C. The pellet was washed three times in sterile water and used to extract the total genomic DNA. DNA samples were extracted using a PowerSoil^®^ DNA isolation kit according to the manufacturer’s instructions. The integrity of the total DNA was then confirmed by 1% agarose gel electrophoresis.

The extracted DNA samples were sequenced with Biomarker Technologies (Beijing, China) using a PacBio Sequel platform (Pacific Biosciences, Menlo Park, CA, United States). The primers 27F (5′-AGRGTTTGATYNTGGCTCAG-3′) and 1492R (5′-TASGGHTACCTTGTTASGACTT-3′) were used to amplify the full-length 16S ribosomal RNA (rRNA) gene sequences. PCR primers targeting full-length 18S rRNA were Euk-A (5′-AACCTGGTTGATCCTGCCAGT-3′) and Euk-B (5′-GATCCTTCTGCAGGTTCACCTAC-3′). PCR amplification was conducted under the following cycling conditions: an initial denaturation at 95°C for 5 min, followed by 25 cycles at 95°C for 30 s, 50°C for 30 s, and 72°C for 1 min, and a final 7 min extension at 72°C.

Raw data were extracted and filtered to obtain the circular consensus sequencing (CCS) sequence using SMRT Link v8.0 (minimum passes ≥5, minimum predicted accuracy ≥0.9). The barcode reads were recognized using Lima v1.7.0 software to acquire raw barcode-CCS sequence data. Then, the raw barcode-CCS sequence data were tested, and chimeric reads were removed using UCHIME v.8.1 to obtain the optimized sequence (Edgar et al., 2011). The sequences were classified into OTUs based on 97% threshold identity using USEARCH v.10.0 software (Edgar, 2013). Then, representative sequences were compared using the Silva (Release 132) database for 16S rRNA gene sequences and Unite (Release 8.1) database for 18S rRNA gene sequences to obtain classified information. These sequence data have been submitted to the NCBI under the accession number PRJNA764352 (for bacteria) and PRJNA764359 (for fungi), respectively.

### Statistical Analyses

The difference was analyzed by one-way ANOVA using R software, and the significance was defined as a *p*-value of less than 0.05. A hollow pie chart at the genus level and bar-plot at the species level of bacterial and fungal communities were produced using sigma-plot 12.5 software. Pearson correlation coefficient was used to analyze the relationship between BA content and microbial community of oat silage, and a heatmap was generated using R software by pheatmap package. Alpha diversity, including abundance-based coverage estimation (ACE), Chao1, and Shannon indices, were calculated using Mothur (v.1.30). A simple network analysis of shared and unique genera (with >1% relative abundance) among the nine different oat silage treatments was performed using Gephi software (0.9.2).

## Results

### Biogenic Amine Content in Oat Forage Before Ensiling

The BA content the of oat forage before ensiling is summarized in Table 1. Eight BAs were analyzed, including tryptamine, β-phenethylamine, putrescine, cadaverine, histamine, tyramine, spermidine, and spermine. Only 10.6 mg/kg dry matter (DM) of putrescine was detected in oat forage, while tryptamine, β-phenylethylamine, cadaverine, histamine, tyramine, spermidine, and spermine were not detected.

**TABLE 1:** The biogenic amine (BA) contents in oat forage before ensiling (mg/kg DM).

**Item**	**Mean value**	**Standard error**
Tryptamine	ND	NA
β-phenylethylamine	ND	NA
Putrescine	10.6	0.571
Cadaverine	ND	NA
Histamine	ND	NA
Tyramine	ND	NA
Spermidine	ND	NA
Spermine	ND	NA

*DM, dry matter; ND, not detected; NA, not available.*

### Fermentation Quality Property and Biogenic Amine Content in Terminal and Aerobically Exposed Oat Silage

The fermentation quality properties of the terminal silage and aerobically exposed oat silage are shown in Table 2. For terminal oat silage, except for the control, the pH values of P and SB silages declined below 4.5. The addition of P and SB increased the lactic acid content (*p* < 0.001) and decreased the ammonia nitrogen content in oat silage (*p* < 0.001). No butyric acid was detected in the P and SB silages, while 51.3 g/kg DM of butyric acid was detected in the control silage. After 7 days of aerobic exposure, the P silage had the highest lactic acid level and the lowest acetic acid level among all treatments (*p* < 0.01). After 14 days of aerobic exposure, the pH and ammonia nitrogen content of P silage increased, while the contents of lactic acid, acetic acid, and propionic acid dramatically decreased. Butyric acid was not detected in the aerobically exposed oat silage.

**TABLE 2:** The fermentation quality properties and BA contents in terminal and aerobically exposed oat silage.

**Item**	**Terminal silage**			**SEM**	***p*-value**	**7 days of aerobic exposure**			**SEM**	***p-*value**	**14 days of aerobic exposure**			**SEM**	***p-*value**
	**Con**	**P**	**SB**			**Con**	**P**	**SB**			**Con**	**P**	**SB**		
pH value	5.92^a^	4.28^c^	4.46^b^	0.260	<0.001	5.94^a^	4.39^b^	4.38^b^	0.287	<0.001	6.02^b^	8.89^a^	4.49^c^	0.728	<0.001
Lactic acid (g/kg DM)	7.1^c^	112.7^a^	83.3^b^	15.80	<0.001	7.5^c^	100.6^a^	82.2^b^	14.25	<0.001	8.7^b^	8.8^b^	90.9^a^	15.09	<0.001
Acetic acid (g/kg DM)	33.2	15.5	24.0	3.252	0.057	35.6^a^	7.7^b^	23.4^a^	4.521	0.008	26.3	2.0	23.1	5.529	0.146
Propionic acid (g/kg DM)	26.9	50.7	8.4	12.76	0.417	15.5	32.0	8.2	6.251	0.321	13.1	4.2	9.3	2.280	0.314
Butyric acid (g/kg DM)	51.3^a^	ND^b^	ND^b^	8.697	<0.001	39.1^a^	ND^b^	ND^b^	7.302	0.009	35.9^a^	ND^b^	ND^b^	8.099	0.094
AN (g/kg TN)	101.0^a^	29.7^b^	20.6^b^	13.07	<0.001	114.6^a^	34.9^b^	23.5^b^	14.63	<0.001	126.6^a^	86.8^b^	26.7^c^	14.85	<0.001
Try (mg/kg DM)	10.3	ND	ND	2.584	0.176	0.7	ND	ND	0.220	0.422	ND	ND	ND	/	/
Phe (mg/kg DM)	37.8^a^	ND^b^	ND^b^	7.053	0.008	37.7^a^	ND^b^	ND^b^	7.026	0.008	33.4^a^	ND^b^	ND^b^	5.937	0.002
Put (mg/kg DM)	619.0^a^	411.7^b^	386.2^b^	39.55	0.002	635.4^a^	218.7^c^	323.1^b^	65.56	0.001	569.9^a^	ND^c^	354.2^b^	83.47	<0.001
Cad (mg/kg DM)	895.6^a^	117.3^c^	342.8^b^	117.2	<0.001	959.4^a^	63.8^c^	287.6^b^	135.6	<0.001	840.9^a^	ND^c^	317.2^b^	123.9	<0.001
His (mg/kg DM)	47.2^a^	ND^b^	ND^b^	7.904	<0.001	38.7^a^	ND^b^	ND^b^	6.478	<0.001	29.4^a^	ND^b^	ND^b^	4.905	<0.001
Tyr (mg/kg DM)	896.9^a^	697.6^b^	331.8^c^	83.54	<0.001	902.4^a^	479.7^b^	299.2^b^	95.10	0.002	809.5^a^	ND^c^	311.2^b^	118.0	<0.001
Spd (mg/kg DM)	ND	ND	ND	NA	NA	ND	ND	ND	NA	NA	ND	ND	ND	NA	NA
Spm (mg/kg DM)	ND	ND	ND	NA	NA	ND	ND	ND	NA	NA	ND	ND	ND	NA	NA
TBA (mg/kg DM)	2506.7^a^	1226.5^b^	1060.8^b^	231.37	<0.001	2574.3^a^	762.2^b^	910.0^b^	296.67	<0.001	2283.1^a^	ND^c^	982.5^b^	331.7	<0.001

*DM, dry matter; AN, ammonia nitrogen; TN, total nitrogen; Try, tryptamine; Phe, β-phenylethylamine; Put, putrescine; Cad, cadaverine; His, histamine; Tyr, tyramine; Spd, spermidine; Spm, spermine; TBA, total biogenic amine; Con, control; P, propionic acid treatment; SB, sodium benzoate treatment; ND, not detected; NA, not available.*

*Within a row, means with different superscripts (a, b, and c) differ significantly from each other (*p* < 0.05).*

The BA content of terminal oat silage differed after treatment with the two additives (Table 2). Putrescine, cadaverine, and tyramine were the dominant BAs produced in oat silage. Spermine and spermidine were not detected in any oat silage. Tryptamine, β-phenylethylamine, and histamine were detected at low levels in control but were not detected in the P and SB silages. Control silage had the highest content of total biogenic amines (TBA) (2506.7 mg/kg DM), as well as the highest content of putrescine (619.0 mg/kg DM), cadaverine (895.6 mg/kg DM), and tyramine (896.9 mg/kg DM) among three treatments. The addition of P and SB resulted in a decrease in putrescine, cadaverine, tyramine, and TBA (*P* < 0.01).

After 7 and 14 days of aerobic exposure, the contents of putrescine, cadaverine, and tyramine in P and SB silages were significantly lower than those in the control (*P* < 0.01). The content of tryptamine, β-phenylethylamine, putrescine, cadaverine, histamine, tyramine, and TBA in oat silage decreased with aerobic exposure. After 7 and 14 days of aerobic exposure, the putrescine, cadaverine, tyramine, and TBA contents of P silage were lower than those at the beginning of silage opening. After 14 days of aerobic exposure, eight BAs were not detected in the P silage.

### Temperature Dynamic of Oat Silage During Aerobic Exposure

**FIGURE 1:**
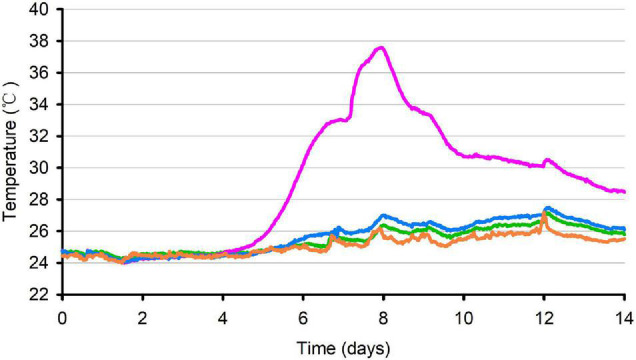
Temperature of oat silage during aerobic exposure. Average temperature (°C) in control (*green*), P silage (*pink*), SB silage (*blue*) and ambient temperature (*orange*) were recorded during 14 days of aerobic exposure.

The temperature dynamics of oat silage during 14 days of aerobic exposure are shown in Figure 1. After 128.5 h of aerobic exposure, the temperature of P silage exceeded the ambient temperature of 2°C. After 191 h of aerobic exposure, the temperature of P silage reached its peak and exceeded the ambient temperature of 11.7°C, and since then, the temperature of P silage began to decline, but it was always higher than the ambient temperature of 2°C. The control and SB silages remained stable during 14 days of aerobic exposure and never exceeded the ambient temperature of 2°C (*p* < 0.05).

### Alpha Diversity of Microbial Community in Terminal and Aerobically Exposed Oat Silage

Alpha diversity was evaluated using Mothur (version v.1.30), and the results are shown in Table 3. Good’s estimator of coverage was 0.99 for all oat silage samples. In terminal silage and aerobically exposed silages, the Shannon, ACE, and Chao1 indices of bacteria in P silage were lower than those in control and SB silage; the lowest Shannon index of fungi was found in P silage. After 7 and 14 days of aerobic exposure, the ACE and Chao1 indices of fungi in P silage were lower than those in control and SB silages.

**TABLE 3:** Alpha diversity of microbial in terminal and aerobically exposed oat silage.

**Item**		**Terminal silage**			**SEM**	***p*-value**	**7 days of aerobic exposure**			**SEM**	***p*-value**	**14 days of aerobic exposure**			**SEM**	***p*-value**
		**Con**	**P**	**SB**			**Con**	**P**	**SB**			**Con**	**P**	**SB**		
Bacteria	Shannon	2.50^a^	1.38^b^	2.60^a^	0.197	<0.001	2.45^a^	1.77^b^	2.70^a^	0.147	0.001	2.72	1.77	2.61	0.213	0.096
	ACE	82.2	64.6	119.2	10.72	0.082	73.4^b^	52.5^b^	132.2^a^	13.14	0.006	103.2^a^	50.0^b^	96.1^a^	8.829	0.001
	Chao1	85.6	62.5	118.9	10.55	0.063	76.1^b^	46.6^b^	138.6^a^	14.47	0.002	111.3^a^	43.5^b^	93.6^a^	10.88	0.002
	Good’s	0.99	0.99	0.99	0.001	0.168	0.99	0.99	0.99	0.001	0.676	0.99	0.99	0.99	0.0003	0.050
Fungi	Shannon	2.70^a^	0.34^c^	1.44^b^	0.375	0.005	2.28^a^	0.08^b^	2.47^a^	0.011	0.011	2.37	0.68	2.72	0.231	0.069
	ACE	107.0	80.2	76.7	19.34	0.828	170.4^a^	16.0^b^	134.4^a^	0.004	0.004	69.3^b^	65.1^b^	165.1^a^	20.12	0.040
	Chao1	151.1	61.0	81.4	10.39	0.765	170.4^a^	16.5^a^	134.7^b^	0.005	0.005	81.6^b^	53.5^b^	171.0^a^	19.86	0.009
	Good’s	0.99	0.99	0.99	0.001	0.422	0.99	0.99	0.99	0.002	0.294	0.99	0.99	0.99	0.001	0.155

*Con, control; P, propionic acid treatment; SB, sodium benzoate treatment.*

*Within a row, means with different superscripts (a, b, and c) differ significantly from each other (*p* < 0.05).*

### Bacterial Community in Terminal and Aerobically Exposed Oat Silage

**FIGURE 2:**
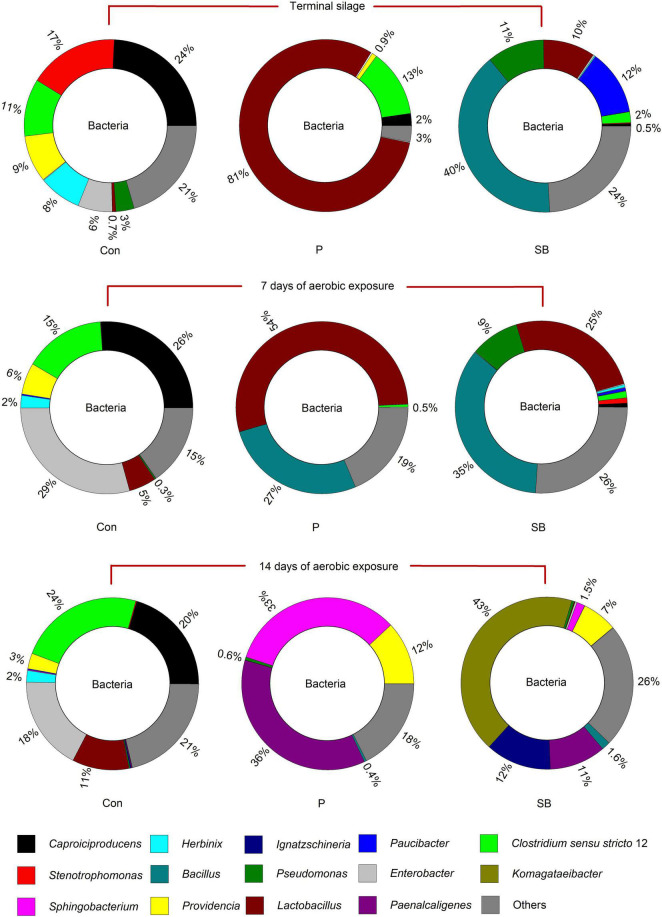
Relative abundance of bacterial community at the genus level in oat silage.

The relative abundance of the bacterial community at the genus level in the terminal and aerobically exposed oat silage is shown in Figure 2. At the end of ensiling, *Caproiciproducens*, *Stenotrophomonas*, *Providencia*, *Herbinix*, and *Enterobacter* were predominant in the control, accounting for 24, 17, 9, 8, and 6% of the total genus, respectively; the relative abundance of *Lactobacillus* increased from 0.7 to 81%, while that of *Caproiciproducens* decreased from 24 to 2% when propionic acid was applied; the dominant bacteria of SB silage were *Bacillus*, *Pseudomonas*, *Lactobacillus*, and *Paucibacter*, at 40, 11, 10, and 12%, respectively. After 7 and 14 days of aerobic exposure, *Lactobacillus*, *Enterobacter*, and *Clostridium sensu stricto* 12 were the dominant genera in the control, and the bacterial community flora of the control was relatively more stable than that of the P and SB silages. After 14 days of aerobic exposure, *Sphingobacterium* (33%), *Paenalcaligenes* (36%), and *Providencia* (12%) were the dominant bacteria in P silage, while *Komagataeibacter* (43%), *Ignatzschineria* (12%), *Paenalcaligenes* (11%), and *Providencia* (7%) were the most abundant bacteria in the SB silage.

**FIGURE 3:**
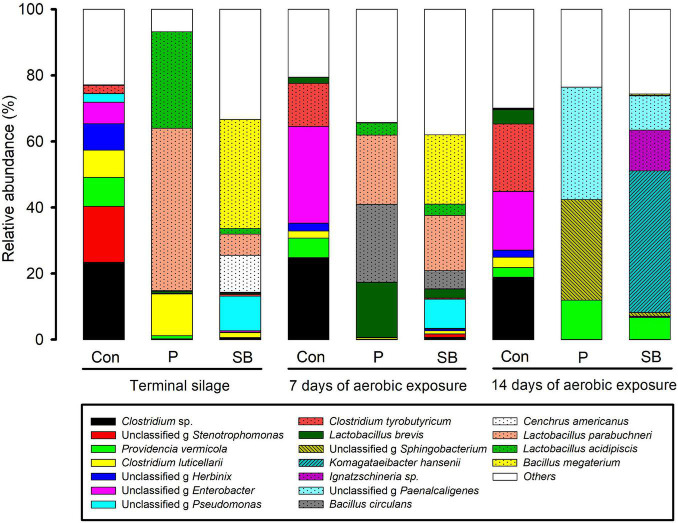
Relative abundance of bacterial community at the species level in oat silage.

The relative abundance of the bacterial community at the species level in the terminal and aerobically exposed oat silage are shown in Figure 3. At the end of ensiling, the relative abundance of *Clostridium* sp. and *Providencia vermicola* in the control were higher than in P and SB silages. *Lactobacillus parabuchneri* and *Lactobacillus acidipiscis* were abundant in P silage, while *Bacillus megaterium*, *Lactobacillus parabuchneri*, and *Cenchrus americanus* were the dominant bacteria in the SB silage. After 7 and 14 days of aerobic exposure, *Clostridium* sp. and *Clostridium tyrobutyricum* were dominant in the control. *Lactobacillus brevis*, *Lactobacillus parabuchneri*, and *Bacillus circulans* were dominant in the P silage after 7 days of air exposure, while *Providencia vermicola* was the dominant bacterium after 14 days of aerobic exposure. In SB silage, *Komagataeibacter hansenii* was the most abundant species, followed by *Ignatzschineria sp.* and *Providencia vermicola*, after 14 days of aerobic exposure.

**FIGURE 4:**
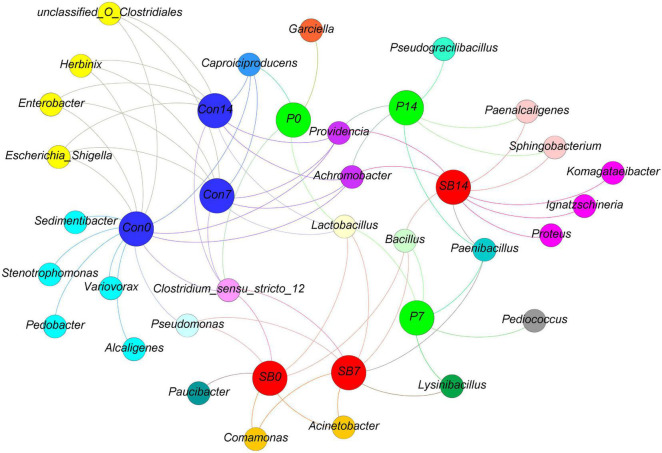
Distribution of shared and unique bacterial genera in oat silage as represented by a simple network analysis. Each treatment of control, P silage, SB silage are represented in blue, green, and red circles, respectively, while shared or unique bacterial genera are represented in smaller circles. Con0, control of terminal silage; Con7, control of 7 days of aerobic exposure; Con14, control of 14 days of aerobic exposure; P0, propionic acid treatment of terminal silage; P7, propionic acid treatment of 7 days of aerobic exposure; P14, propionic acid treatment of 14 days of aerobic exposure; SB0, sodium benzoate treatment of terminal silage; SB7, sodium benzoate treatment of 7 days of aerobic exposure; SB14, sodium benzoate treatment of 14 days of aerobic exposure.

We observed some bacterial genera unique to each silage, according to the analysis of shared OTU contents among all silages (Figure 4). The genera *Stenotrophomonas*, *Sedimentibacter*, *Pedobacter*, *Variovorax*, and *Alcaligenes* were unique to the terminal Con0 silage. The genus *Garciella* was found only in terminal P0 silage, and the abundance of *Garciella* in P0 was significantly higher than Con0 and SB0 (Supplementary Table 1, *p* < 0.05). The genus *Paucibacter* appeared only in the terminal SB0 silage. The genus *Pseudogracilibacillus* was found only in the P14 silage. The genera *Komagataeibacter*, *Ignatzschineria*, and *Proteus* were unique to the SB14 silage.

### Fungal Community in Terminal and Aerobically Exposed Oat Silage

**FIGURE 5:**
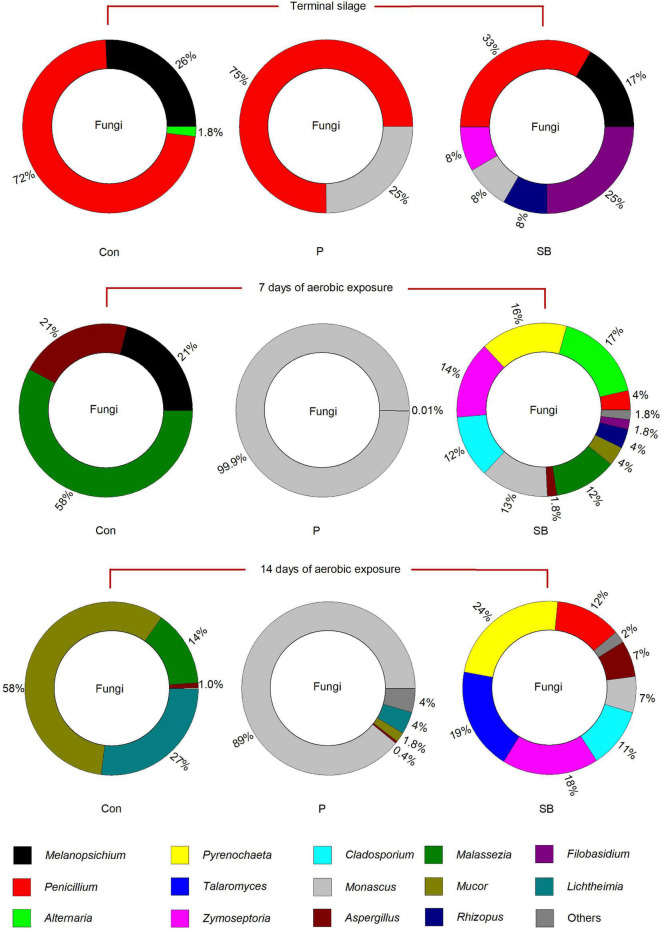
Relative abundance of fungal community at the genus level in oat silage.

The relative abundance of fungal communities at the genus level in terminal and aerobically exposed oat silage is shown in Figure 5. At the end of ensiling, the fungal community involved in the control consisted predominantly of the genera *Penicillium* and *Melanopsichium*. *Penicillium* and *Monascus* were the predominant fungi in P silage, while *Melanopsichium*, *Penicillium*, and *Filobasidium* were the predominant fungi in SB-treated silages. After aerobic exposure, the relative abundance of *Monascus* increased in the P silage and was much higher than that in the control and SB silages.

**FIGURE 6:**
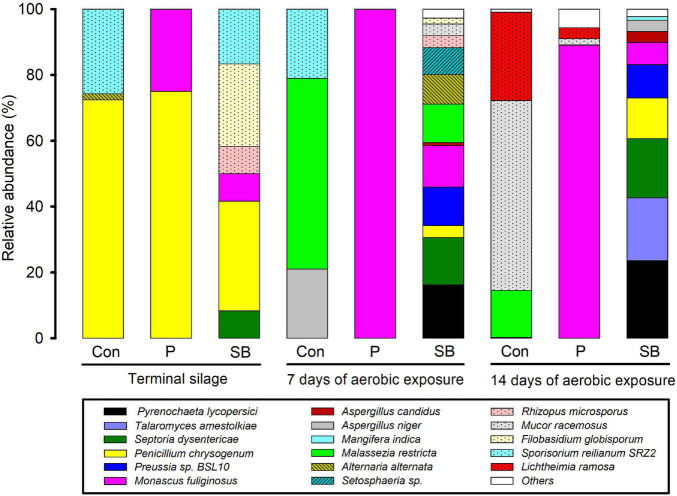
Relative abundance of fungal community at the species level in oat silage.

The relative abundance of fungal communities at the species level in terminal and aerobically exposed oat silage are shown in Figure 6. At the end of ensiling, *Penicillium chrysogenum* was the dominant species in control, P, and SB silages. After 7 and 14 days of aerobic exposure, *Monascus fuliginosus* was the dominant species in P silage, while *Pyrenochaeta lycopersici*, *Septoria dysentericae*, *Preussia* sp. BSL-10, and *Monascus fuliginosus* were the dominant species in the SB silage.

**FIGURE 7:**
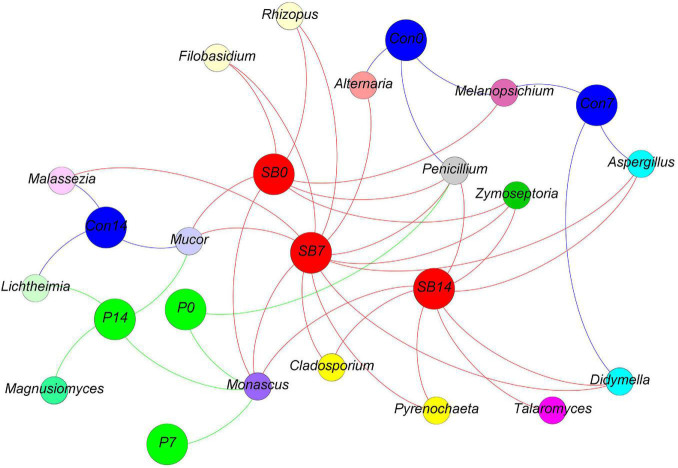
Distribution of shared and unique fungal genera in oat silage as represented by a simple network analysis. Each treatment of control, P silage, SB silage are represented in blue, green, and red circles, respectively, while shared or unique fungal genera are represented in smaller circles. Con0, control of terminal silage; Con7, control of 7 days of aerobic exposure; Con14, control of 14 days of aerobic exposure; P0, propionic acid treatment of terminal silage; P7, propionic acid treatment of 7 days of aerobic exposure; P14, propionic acid treatment of 14 days of aerobic exposure; SB0, sodium benzoate treatment of terminal silage; SB7, sodium benzoate treatment of 7 days of aerobic exposure; SB14, sodium benzoate treatment of 14 days of aerobic exposure.

We observed some fungal genera unique to each silage, according to the analysis of shared OTU contents among all silages (Figure 7). The genus *Magnusiomyces* is unique to the P14 silage. The genus *Talaromyces* is unique to the SB14 silage. The genus *Monascus* was shared in P and SB silages but not present in the control silage.

### Correlation Relationship Between Microbial Community and Biogenic Amine

**FIGURE 8:**
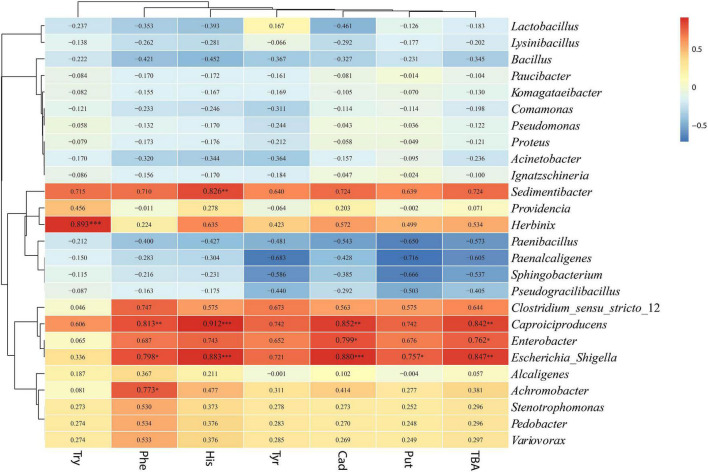
Correlation analysis of the bacterial community with BA at the genus level. ^∗^*p* < 0.05, ^∗∗^*p* < 0.01, and ^∗∗∗^*p* < 0.001, respectively.

**FIGURE 9:**
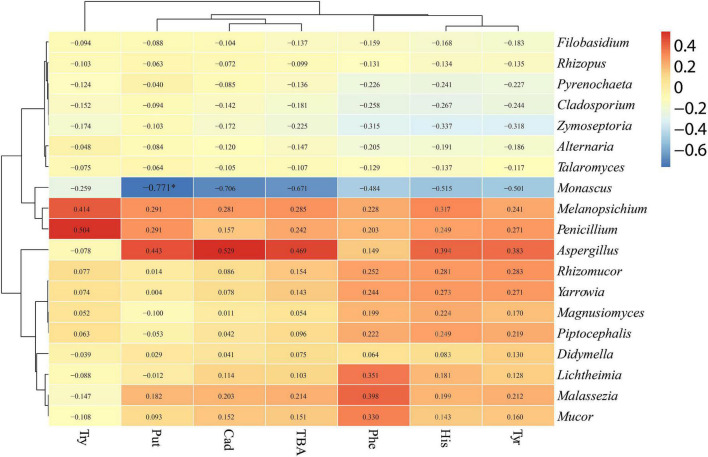
Correlation analysis of the fungal community with BA at the genus level. ^∗^*p* < 0.05.

The relationships between bacterial and fungal communities and BA were evaluated using Pearson analysis. For bacteria, the genus *Caproiciproducens* showed significant positive correlations with β-phenylethylamine (*R* = 0.813, *p* < 0.01), histamine (*R* = 0.912, *p* < 0.001), cadaverine (*R* = 0.852, *p* < 0.01), and total biogenic amine (*R* = 0.842, *p* < 0.01); the genus *Escherichia-Shigella* showed significant positive correlations with β-phenylethylamine (*R* = 0.798, *p* < 0.05), histamine (*R* = 0.883, *p* < 0.001), cadaverine (*R* = 0.880, *p* < 0.001), putrescine (*R* = 0.757, *p* < 0.05), and total biogenic amine (*R* = 0.847, *p* < 0.01); the genus *Enterobacter* showed a significantly positive correlation with cadaverine (*R* = 0.799, *p* < 0.05) and TBA (*R* = 0.762, *p* < 0.05) (Figure 8). For fungi, *Monascus* showed a significantly negative correlation with putrescine at the genus level (*R* = −0.771, *p* < 0.05) (Figure 9).

## Discussion

### Fermentation Quality Property and Biogenic Amine Content in Terminal and Aerobically Exposed Oat Silage

Silage pH is a vital parameter indicating the degree of fermentation, and a lower pH ensures better anaerobic fermentation and further inhibits undesirable microorganisms (Pahlow et al., 2003). The production of organic acid in silage results in the decrease of pH, while the formation of ammonia nitrogen and amines results in the increase of pH (McDonald et al., 1991). In this study, a significant decrease in pH was observed in P and SB silages, which might be mainly caused by the significant increase of lactic acid. Lactic acid is the strongest acid among all silage organic acids and its presence will decrease pH more efficient than other volatile acid. The high pH of control silage might be due to the considerable accumulation of basic nitrogenous substances such as ammonia and amines. Besides, high level of butyric acid (51.3 g/kg DM) was detected in the control. The presence of butyric acid in silage is undesirable; >5 g/kg DM of butyric acid indicates that proteolysis in the control was extensive, and the substantial clostridial activity was not inhibited (Muck, 2010).

BA is an endogenous metabolic constituent of cells in plants (Halász et al., 1994). Among the eight BAs measured in this study, only 10.6 mg/kg DM of putrescine was detected in oat forage. This was consistent with Nishino et al. (2007), who observed a low putrescine content (11.0 mg/kg DM) in fresh maize material. BA has been widely detected in various silages, such as alfalfa, Italian ryegrass, maize, oat, orchardgrass, red clover, and total mixed ration (Křížek, 1993; Steidlová and Kalač, 2002; Nishino et al., 2007; Selwet et al., 2013; Scherer et al., 2019). In this study, putrescine, cadaverine and tyramine were the primary BA formed in oat silage, and it was consistent with the results for maize silage reported by Steidlová and Kalač (2002) and Nishino et al. (2007). The large production of putrescine, cadaverine, and tyramine in silage are partly due to their precursor amino acids, arginine, lysine, and tyrosine, respectively (Joosten and Northolt, 1987). Histamine was detected at low levels in oat silage, while high amounts of histamine were detected in alfalfa silage (Selwet et al., 2013) and ryegrass silage (Nishino et al., 2007). Spermidine and spermine were not detected in any oat silage samples but were found in maize silage (Steidlová and Kalač, 2002) and orchard grass silage (Křížek, 1993). The differences in the type and content of BA in silage may be due to the variations in forage materials, microbial communities, and fermentation conditions (Scherer et al., 2015).

Moreover, in the present study, the control silage had the highest content of ammonia nitrogen (101.0 g/kg TN) and TBA (2506.7 mg/kg DM), as well as the highest content of tryptamine (10.3 mg/kg DM), β-phenylethylamine (37.8 mg/kg DM), putrescine (619.0 mg/kg DM), cadaverine (895.6 mg/kg DM), histamine (47.2 mg/kg DM), and tyramine (896.9 mg/kg DM) among all treatments. McDonald et al. (1991) suggested that poorly fermented silages contain a high content of amines (>2000 mg/kg DM), which may be associated with losses in feeding value and low palatability. In comparison, the content of the TBA in control silage appeared to exceed 2000 mg/kg DM. The high levels of BA and ammonia nitrogen indicated progressive deamination and decarboxylation activity during fermentation in the control silage. The BA content in oat silage differed after treatment with chemical additives; TBA decreased by 51.1 and 57.7% in P and SB silages, respectively. These results suggest that P and SB could retard the formation of putrescine, cadaverine, and tyramine in oat silage, which may contribute to the low pH of P and SB silages, and the acidic environment limits microbial decarboxylation (McDonald et al., 1991).

After aerobic exposure, the ammonia nitrogen content increased in oat silage, whereas the organic acid and BA contents decreased. This phenomenon was more evident in P silage, the content of ammonia nitrogen increased to 86.8 g/kg TN, while the organic acid content decreased dramatically, and BA was not detected in P silage after 14 days of aerobic exposure. It is hypothesized that microorganisms may utilize organic substance (BA and organic acids) in oat silage after aerobic exposure. The high ammonia nitrogen content in aerobically exposed oat silage indicates that the deamination increased after air exposure, which was related to the pH increase in aerobically exposed oat silage.

### Aerobic Stability of Oat Silage

Previous studies have documented that the application of 0.2–0.4% propionic acid-based additives could improve the stability of silage (Chen et al., 2014; Zhang et al., 2018). The increase in pH and temperature and loss of lactic acid in P silage indicate that propionic acid reduced rather than improved aerobic stability; this result is in line with the results of He et al. (2020b). In contrast, SB treatment effectively controlled aerobic deterioration by minimizing the rise in pH and temperature. Oliveira et al. (2017) reported that decreasing antifungal agents, such as acetic acid, would result in aerobic deterioration. We did observe a lower content of acetic acid and a higher content of lactic acid in P silage than in control and SB silage. The stabilization of temperature in the control and SB silages may correlate with the high acetic acid content. Moreover, the high content of butyric acid may also cause the steadiness of temperature in the control. Danner et al. (2003) and Zhang et al. (2018) observed that the application of butyric acid improved the aerobic stability of silage. Similar results were documented by Li et al. (2016), who reported that rice straw silage without additive treatment presented higher butyric acid content and aerobic stability.

### Bacterial Community in Terminal and Aerobically Exposed Oat Silage

At the end of ensiling, the bacterial community in the control was mainly represented by the genera *Caproiciproducens*, *Stenotrophomonas*, *Providencia*, *Herbinix*, and *Enterobacter*, accounting for 24, 17, 9, 8, and 6% of the total genera, respectively. Among them, the *Caproiciproducens* and *Herbinix* both belong to the order Clostridiales of class Clostridia. Clostridia may cause extensive protein degradation and butyric acid accumulation in silage (Wang et al., 2019). *Clostridium* sp. (genus *Caproiciproducens*) was the most abundant species in control oat silage. This was consistent with the finding of Ni et al. (2020), in which the control alfalfa silage exhibited higher rRNA copy numbers of *Clostridium* sp. In addition, both *Providencia* and *Enterobacter* are genera of Gram-negative bacteria in Enterobacteriaceae family and are associated with some diseases in animals (Gupta et al., 2012; Patel et al., 2016). The growth of *Enterobacter* during silage is related to the production of ammonia nitrogen and acetic acid (Muck, 2010; Ni et al., 2017). The genus *Stenotrophomonas* was also found in Alfalfa silage (Ogunade et al., 2018), corn stalk silage (He et al., 2020a), and *Pennisetum sinese* silage (Zi et al., 2021), and the proteolytic activity is universal in *Stenotrophomonas* (Hayward et al., 2010).

The bacterial communities were significantly affected by the addition of P and SB; the abundance of *Caproiciproducens*, *Stenotrophomonas*, *Providencia*, *Herbinix*, and *Enterobacter* decreased after treatment with P and SB. The abundance of *Lactobacillus* in the P and SB silages was higher than that in the control in the terminal silage, which is consistent with the low pH in P and SB silages. Besides, the abundance of *Lactobacillus* in P silage (81%) was higher than that in the control (0.7%) and SB silage (10%), which is consistent with a previous study showing that the addition of propionic acid can promote the growth of lactic acid bacteria (Papendick and Singh-Verma, 1972). In addition, *Bacillus megaterium* was the most dominant population in the SB silage. *Bacillus megaterium* is a facultative anaerobic Gram-positive bacterium that can secrete antimicrobial compounds (Rajabi et al., 2020) and is widely used as a probiotic inoculant in the silage and feed industry (Deng et al., 2021). Some *Bacillus* species could increase the lactic acid concentration and improve the aerobic stability of silage (Lara et al., 2016; Bai et al., 2020) by producing antimicrobial substances. Thus, the high abundance of *Bacillus* may be responsible for the improvement of fermentation quality and aerobic stability of SB silage.

After 7 and 14 days of aerobic exposure, the abundance of genera *Clostridium sensu stricto* 12, *Enterobacter*, and *Lactobacillus* in the control increased, while that of *Stenotrophomonas, Providencia*, and *Herbinix* decreased. *Clostridium sensu stricto* 12 belongs to the order Clostridiales of class Clostridia (McDonald et al., 1991). Although Clostridia are strictly anaerobic, studies have shown that the growth of Clostridia can occur during aerobic exposure (Pahlow et al., 2003). In the present study, the abundance of *Clostridium tyrobutyricum* in the control silage increased after exposure to air. This result is consistent with the study by Jonsson (1991); after 14 days of air exposure, *Clostridium tyrobutyricum* spores largely increased. A possible explanation for *Clostridium tyrobutyricum* growth during aerobic exposure is that aerobic and anaerobic riches coexist in the silage and that Clostridia profit from the oxidation of the preserving acid by aerobic organisms (McDonald et al., 1991). After 7 days of aerobic exposure, the bacterial communities of the P and SB silages were not significantly altered. After 14 days of aerobic exposure, the structure of bacterial communities in P and SB silages completely changed; the genera *Sphingobacterium* and *Paenalcaligenes* were dominant in P silage, while *Komagataeibacter*, *Ignatzschineria*, and *Paenalcaligenes* were the predominant genera in SB silage. These bacteria have rarely been reported in silage.

Among the unique genera detected in terminal control silage, *Stenotrophomonas* and *Sedimentibacter* are proteolytic/amino acid-utilizing bacteria (Breitenstein et al., 2002; Hayward et al., 2010). The genus *Alcaligenes* has been reported to produce a larger amount of BA in meat (Lakshmanan et al., 2002). *Garciella* (Clostridia class) was found only in terminal P silage and is an undesirable bacterium in silage (Zheng et al., 2020). *Paucibacter* appeared only in terminal SB silage, and it has rarely been reported in previous ensilage research. The genus *Pseudogracilibacillus* was found only in the P14 silage. Previous studies reported that *Pseudogracilibacillus* appeared in the composting process and was able to degrade organic matter (Awasthi et al., 2020). This was in accordance with those of the present study, in which the P14 silage had the lowest organic acid and BA contents.

### Fungal Community in Terminal and Aerobically Exposed Oat Silage

Information about epiphytic fungal communities in silage is scarce, although the presence and production of mycotoxins have been widely studied. The fungi present in this study mainly belong to the genera *Melanopsichium*, *Penicillium*, *Alternaria*, *Pyrenochaeta*, *Talaromyces*, *Zymoseptoria*, *Cladosporium*, *Monascus*, *Aspergillus*, *Malassezia*, *Mucor*, *Rhizopus*, *Filobasidium*, and *Lichtheimia*. In terminal oat silage, *Penicillium chrysogenum* was the predominant species in the control, P, and SB silages, which are different from the dominant fungal species of *Candida glabrata*, unclassified_g_*Candida* and *Monascus purpureus* reported in corn silage (Bai et al., 2021), and the predominant fungi of *Pichiaceae*, *Trichocomaceae*, and *Debaryomycetaceae* reported in oat silage (Romero et al., 2017). The *Penicillium chrysogenum* was mainly found in cereals and cheese (Rundberget et al., 2004), while it was rarely reported in silage (O’Brien et al., 2008). The different fungal communities among the studies may be related to the different fermentation processes of ensiling or fungal communities from the soil.

The predominant fungi of the three oat silages were all significantly altered after aerobic exposure, which was in accordance with the previous report (Li and Nishino, 2011). *Monascus fuliginosus* belonging to the family Aspergillus, is an acidophilic fungus that is especially fond of lactic acid (Li, 1982). It can grow under aerobic conditions and survive in a pH range of 3–5 (Yang, 2008). After aerobic exposure, the *Monascus fuliginosus* occupied the fungi community in P silage, which was probably related to the high lactic acid content and low pH environment of P silage. Moreover, the growth of *Monascus* might cause aerobic spoilage in P silage, which was consistent with the findings of He et al. (2020b); the addition of propionic acid increased the abundance of *Monascus* and decreased the aerobic stability of rice straw silage.

We observed some fungal genera unique to each silage, according to the analysis of shared OTU contents among all silages. The genus *Magnusiomyces* was unique to the P14 silage. *Magnusiomyces* is a human pathogen often associated with contaminated dairy products (Gurgui et al., 2011). The genus *Talaromyces* was unique to the SB14 silage. *Talaromyces* was also reported by Wambacq et al. (2016) in grass and whole-crop corn. However, there have been no reports describing the observation of *Magnusiomyces* and *Talaromyces* in silage.

### Correlation Relationship Between Microbial Community and Biogenic Amine in Silage

As mentioned previously, BA formation is associated with the microbial growth (Halász et al., 1994). The correlation between BA and microbial community was evaluated by the Pearson correlation heatmap. In this study, bacterial genera such as *Sedimentibacter*, *Herbinix*, *Caproiciproducens*, *Enterobacter*, *Escherichia_Shigella*, and *Achromobacter* were found to be positively correlated with BA, and fungus *Monascus* was negatively correlated with BA. Among bacteria, the genera *Sedimentibacter*, *Herbinix*, and *Caproiciproducens* belong to order Clostridiales, and the genera *Enterobacter* and *Escherichia_Shigella* belong to order Enterobacteriales. Bacteria of Clostridiales and Enterobacteriales are often present in silage and can break down the protein (McDonald et al., 1991). In addition, *Monascus* showed a significantly negative correlation with putrescine in the present study. Amine oxidase activity was found in the mycelium of *Monascus*, which uses monoamine or diamine as the sole nitrogen source (Schilling and Lerch, 1995). The *Monascus* may degrade the BA in P silage after aerobic exposure.

Tryptamine was significant positively correlated with *Herbinix*; β-phenylethylamine was significant positively correlated with *Caproiciproducens*, *Escherichia_Shigella* and *Achromobacter*; histamine was positively correlated with *Sedimentibacter*, *Caproiciproducens*, and *Escherichia_Shigella;* cadaverine was positively correlated with *Caproiciproducens*, *Enterobacter*, and *Escherichia_Shigella;* and putrescine was positively correlated with *Escherichia_Shigella*. According to the correlation analysis, bacteria *Lactobacillus* and *Bacillus* displayed negative correlation with BA although they have been reported as BA producers in some studies (Suzzi and Gardini, 2003; Mah et al., 2019). A study by Gu et al. (2018) found no apparent correlation between BA content and lactic acid bacteria or other microorganisms in fermented soy curd because biogenic amine formation is highly dependent on bacterial strain rather than microbial species (Beneduce et al., 2010). The bacteria from same genus or species might secrete different types of amino acid decarboxylase (Miladi et al., 2017). Due to the complexity of microbial communities in silage, correlation analysis might not reveal the accurate relationship between BA and microbial communities. The relationship between BA and the microbiome in silage is complicated, and more studies are needed to evaluate the dominant microbial contribution to BA formation in silage. To reflect more precise relationship between microbial and BA, it is necessary to isolate the dominant strain and evaluate their ability to produce amine.

## Conclusion

Significant amounts of putrescine, cadaverine, and tyramine are formed in oat silage, and this formation can be limited by fermentation with propionic acid (P) or sodium benzoate (SB). Both P and SB decreased *Caproiciproducens*, *Stenotrophomonas*, *Herbinix*, and *Enterobacter abundance* and increased *Lactobacillus* abundance in terminal oat silage. However, the P silage exhibited lower aerobic stability than the SB silage. The fungus *Monascus fuliginosus* in aerobically exposed P silage increased the temperature and decreased the quality of oat silage. *Sedimentibacter*, *Herbinix*, *Caproiciproducens*, *Enterobacter*, and *Escherichia-Shigella* may be mainly responsible for the formation of BA in oat silage. In conclusion, the addition of SB could improve fermentation quality and aerobic stability and inhibit BA formation in oat silage.

## Data Availability Statement

The original contributions presented in the study are included in the article/Supplementary Material, further inquiries can be directed to the corresponding author/s.

## Author Contributions

TJ and ZY designed the experiments. TJ and YY conducted the experiments. TJ, YY, and ZY analyzed the data. TJ wrote the manuscript. All authors read and approved the manuscript.

## Conflict of Interest

The authors declare that the research was conducted in the absence of any commercial or financial relationships that could be construed as a potential conflict of interest.

## Publisher’s Note

All claims expressed in this article are solely those of the authors and do not necessarily represent those of their affiliated organizations, or those of the publisher, the editors and the reviewers. Any product that may be evaluated in this article, or claim that may be made by its manufacturer, is not guaranteed or endorsed by the publisher.
