# Supplementary material for: Propionic Acid and Sodium Benzoate Affected Biogenic Amine Formation, Microbial Community, and Quality of Oat Silage
Source: Front Microbiol. 2021 Nov 8;12:750920. doi: 10.3389/fmicb.2021.750920 (PMC8606646; doi:10.3389/fmicb.2021.750920)
Supplement: Supplementary file 1 [file Table_1.DOC]

**Supplementary Table S1. The relative abundance of unique bacteria genera in different treatments.**

| Genera | Con0 | P0 | SB0 | SEM | *P-*value |  | Con7 | P7 | SB7 | SEM | *P-*value |  | Con14 | P14 | SB14 | SEM | *P-*value |
| --- | --- | --- | --- | --- | --- | --- | --- | --- | --- | --- | --- | --- | --- | --- | --- | --- | --- |
| *Stenotrophomonas* | 16.1 | <1.0 | <1.0 | 5.33 | 0.460 |  | <1.0 | <1.0 | <1.0 | NA | NA |  | <1.0 | <1.0 | <1.0 | NA | NA |
| *Paucibacter* | <1.0 | <1.0 | 10.6 | 3.28 | 0.398 |  | <1.0 | <1.0 | <1.0 | NA | NA |  | <1.0 | <1.0 | <1.0 | NA | NA |
| *Komagataeibacter* | <1.0 | <1.0 | <1.0 | NA | NA |  | <1.0 | <1.0 | <1.0 | NA | NA |  | <1.0 | <1.0 | 41.5 | 13.82 | 0.465 |
| *Ignatzschineria* | <1.0 | <1.0 | <1.0 | NA | NA |  | <1.0 | <1.0 | <1.0 | NA | NA |  | <1.0 | <1.0 | 12.8 | 4.06 | 0.428 |
| *Pseudogracilibacillus* | <1.0 | <1.0 | <1.0 | NA | NA |  | <1.0 | <1.0 | <1.0 | NA | NA |  | <1.0 | 5.4 | <1.0 | 1.79 | 0.475 |
| *Proteus* | <1.0 | <1.0 | <1.0 | NA | NA |  | <1.0 | <1.0 | <1.0 | NA | NA |  | <1.0 | <1.0 | 10.0 | 2.98 | 0.387 |
| *Pedobacter* | 1.7 | <1.0 | <1.0 | 0.57 | 0.465 |  | <1.0 | <1.0 | <1.0 | NA | NA |  | <1.0 | <1.0 | <1.0 | NA | NA |
| *Alcaligenes* | 1.4 | <1.0 | <1.0 | 0.46 | 0.484 |  | <1.0 | <1.0 | <1.0 | NA | NA |  | <1.0 | <1.0 | <1.0 | NA | NA |
| *Variovorax* | 1.2 | <1.0 | <1.0 | 0.41 | 0.465 |  | <1.0 | <1.0 | <1.0 | NA | NA |  | <1.0 | <1.0 | <1.0 | NA | NA |
| *Sedimentibacter* | 1.1 | <1.0 | <1.0 | 0.28 | 0.204 |  | <1.0 | <1.0 | <1.0 | NA | NA |  | <1.0 | <1.0 | <1.0 | NA | NA |
| *Garciella* | <1.0^b^ | 1.2^a^ | <1.0^b^ | 0.20 | 0.018 |  | <1.0 | <1.0 | <1.0 | NA | NA |  | <1.0 | <1.0 | <1.0 | NA | NA |
| *Pediococcus* | <1.0 | <1.0 | <1.0 | NA | NA |  | <1.0 | 1.1 | <1.0 | 0.34 | 0.486 |  | <1.0 | <1.0 | <1.0 | NA | NA |

*Con0, control of terminal silage; Con7, control of seven days of aerobic exposure; Con14, control of 14 days of aerobic exposure; P0, propionic acid treatment of terminal silage; P7, propionic acid treatment of seven days of aerobic exposure; P14, propionic acid treatment of 14 days of aerobic exposure; SB0, sodium benzoate treatment of terminal silage; SB7, sodium benzoate treatment of seven days of aerobic exposure; SB14, sodium benzoate treatment of 14 days of aerobic exposure. Within a row, means with different superscripts (a, b) differ significantly from each other (P < 0.05).*
